# Interface Passivation Effects on the Photovoltaic Performance of Quantum Dot Sensitized Inverse Opal TiO_2_ Solar Cells

**DOI:** 10.3390/nano8070460

**Published:** 2018-06-25

**Authors:** Kanae Hori, Yaohong Zhang, Pimsiri Tusamalee, Naoki Nakazawa, Yasuha Yoshihara, Ruixiang Wang, Taro Toyoda, Shuzi Hayase, Qing Shen

**Affiliations:** 1 Department of Engineering Science, The University of Electro Communications, 1-5-1 Chofugaoka, Chofu, Tokyo 182-8585, Japan; hori@jupiter.pc.uec.ac.jp (K.H.); pimsiri.tsml@gmail.com (P.T.); nakazawa@jupiter.pc.uec.ac.jp (N.N.); yoshihara@jupiter.pc.uec.ac.jp (Y.Y.); toyoda@pc.uec.ac.jp (T.T.); 2 Department of Physics, King Mongkut’s Institute of Technology Ladkrabang, 1 Soi Chalongkrung 1, Ladkrabang, Bangkok 10520, Thailand; 3 Beijing Engineering Research Centre of Sustainable Energy and Buildings, Beijing University of Civil Engineering and Architecture, Beijing 102616, China; wangruixiang@bucea.edu.cn; 4 Graduate school of Life Science and Systems Engineering, Kyushu Institute of Technology, 2-4 Hibikino, Wakamatsu-ku, Kitakyushu, Fukuoka 808-0196, Japan; hayase@life.kyutech.ac.jp

**Keywords:** quantum dot-sensitized solar cells (QDSSCs), inverse opal-TiO_2_, surface passivation

## Abstract

Quantum dot (QD)-sensitized solar cells (QDSSCs) are expected to achieve higher energy conversion efficiency than traditional single-junction silicon solar cells due to the unique properties of QDs. An inverse opal (IO)-TiO_2_ (IO-TiO_2_) electrode is useful for QDSSCs because of its three-dimensional (3D) periodic nanostructures and better electrolyte penetration compared to the normal nanoparticles (NPs)-TiO_2_ (NPs-TiO_2_) electrode. We find that the open-circuit voltages *V*_oc_ of the QDSSCs with IO-TiO_2_ electrodes are higher than those of QDSSCs with NPs-TiO_2_ electrodes. One important strategy for enhancing photovoltaic conversion efficiency of QDSSCs with IO-TiO_2_ electrodes is surface passivation of photoanodes using wide-bandgap semiconducting materials. In this study, we have proposed surface passivation on IO-TiO_2_ with ZnS coating before QD deposition. The efficiency of QDSSCs with IO-TiO_2_ electrodes is largely improved (from 0.74% to 1.33%) because of the enhancements of *V*_oc_ (from 0.65 V to 0.74 V) and fill factor (*FF*) (from 0.37 to 0.63). This result indicates that ZnS passivation can reduce the interfacial recombination at the IO-TiO_2_/QDs and IO-TiO_2_/electrolyte interfaces, for which two possible explanations can be considered. One is the decrease of recombination at IO-TiO_2_/electrolyte interfaces, and the other one is the reduction of the back-electron injection from the TiO_2_ electrode to QDs. All of the above results are effective for improving the photovoltaic properties of QDSSCs.

## 1. Introduction

Renewable and low-cost energy is increasingly demanded which has created some remarkable research in the field of next-generation solar cells. Semiconductor quantum dot (QD)-sensitized solar cells (QDSSCs) have attracted much interest because they show some advantages compared to dye-sensitized solar cells [1,2,3,4]. QDs are beneficial because of their high optical absorption coefficients, multiple exciton generation, and large intrinsic dipole moments [4,5,6]. It has been expected that the maximum theoretical efficiency of QD-based solar cells would be 44% [7], which is much higher than that of traditional single-junction silicon solar cells (about 30%) [8].

The morphology of the TiO_2_ electrodes is one main factor for the photovoltaic performance of the QDSSCs. In general, nanoparticles (NPs)-TiO_2_ electrodes are employed in the QDSSCs because they have higher surface areas for adsorbing the QDs, and thus, photocurrents can be enhanced [9,10]. However, the disadvantage of NPs-TiO_2_ electrodes is the low electron transport which results from the interfacial resistance and electron scattering in the NPs electrodes [11]. One dimensional (1D) nanofiber or whisker electrode, which can provide a direct pathway for charge transport, was usually employed in solar cells [12,13]. In addition to the 1D semiconducting oxides, the inverse opal (IO)-TiO_2_ electrode has three dimensional (3D) periodic mesoporous nanostructures, which is also useful for the application of QDSSCs. This is because of the possible better electrolyte penetration in the IO-TiO_2_ electrodes compared to the NPs-TiO_2_ electrodes. In addition, the ordered 3D structure in the case of IO-TiO_2_ electrodes is expected to be better for the electron transport in the TiO_2_ electrode. It was observed that the open-circuit voltage (*V*_oc_) of the CdSe QDSSCs with IO-TiO_2_ electrodes was 0.2 V higher than that of the QDSSCs with the NPs-TiO_2_ electrodes [14,15].

**Figure 1:**
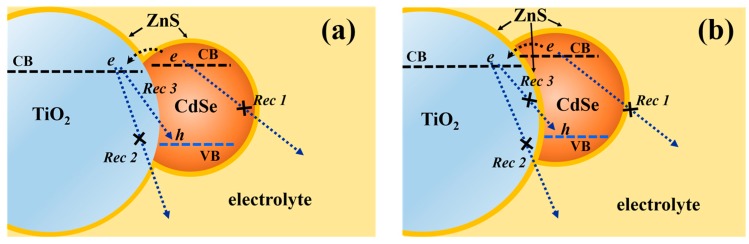
Schematic diagrams of interfacial recombination paths occurring at a photoelectrode. The ZnS coating deposited after the deposition of CdSe quantum dots (QDs) (**a**); and before and after the deposition of CdSe QDs (**b**).

In QDSSCs, the interfacial recombination plays an important role in the performance of QDSSCs, especially in the *V*_oc_ of the device. As shown in Figure 1, there are usually three interfacial recombination paths occurring at the photoelectrode of QDSSCs, i.e., at the QDs/electrolyte interface (Rec 1), at the TiO_2_/electrolyte interface (Rec 2), and at the TiO_2_/QDs interface (Rec 3) [16,17,18]. One important strategy for reducing the recombination, thereby enhancing photovoltaic conversion efficiency of QDSSCs, is the surface passivation of the photoanode using wide-bandgap semiconducting materials. ZnS is commonly used as the surface passivation layer on QD-sensitized photoanodes [9,14,19,20]. ZnS passivation by the successive ionic layer adsorption and reaction (SILAR) method utilizes its advantage of straightforward preparation and striking enhancement of photovoltaic conversion efficiency. Diguna et al. applied CdSe QDSSCs on IO-TiO_2_ electrodes, in which ZnS passivation was applied on the surfaces of CdSe QDs [14]. All of the photovoltaic performances were significantly improved. Hachiya et al. applied the ZnS passivation on the PbS QDs and studied the influences of the ZnS passivation on electron injection and charge recombination processes of the PbS QDSSCs [20]. Their results demonstrated that the ZnS passivation could greatly enhance the charge injection efficiency through the decrease in the carrier trapping and charge recombination (Rec 1 and Rec 2) after the ZnS passivation. However, in those works, the surface passivation of the photoanode by ZnS coating can only efficiently diminish the recombination occurring through the Rec 1 and Rec 2 paths in QDSSCs; thus, the recombination occurring at the TiO_2_/QDs interface (Rec 3) also needs to be blocked. However, there are no reports on the surface passivation of IO-TiO_2_ before the QDs deposition to date.

In this study, we have proposed surface passivation on IO-TiO_2_ with ZnS passivation before QD deposition. Effects of ZnS passivation cycles on the photovoltaic properties have been investigated systematically. Passivation at the interface between IO-TiO_2_ and QDs with ZnS coating improved the fill factor (*FF*) and *V*_oc_ significantly because of the suppression of interfacial recombination, which corresponded to an increase of the electron lifetime in the TiO_2_ electrodes after the ZnS passivation.

## 2. Results and Discussion

**Figure 2:**
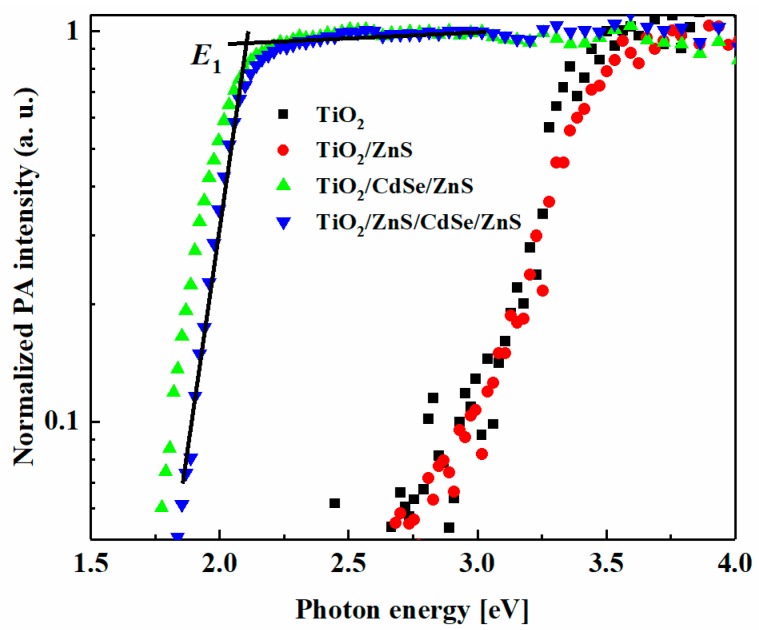
Normalized photoacoustic (PA) spectra of TiO_2_, TiO_2_/ZnS, TiO_2_/CdSe QD/ZnS and TiO_2_/ZnS/CdSe QD/ZnS.

We prepared four different types of samples, i.e., TiO_2_, TiO_2_/ZnS, TiO_2_/CdSe QD/ZnS and TiO_2_/ZnS/CdSe QD/ZnS samples. The former two were IO-TiO_2_ electrodes with and without ZnS passivation on the electrodes. The latter two were CdSe QDs adsorbed on these IO-TiO_2_ electrodes, and finally, ZnS (2 cycles) were adsorbed on the QD surfaces. The optical absorption properties of the prepared electrodes were investigated using the photoacoustic (PA) technique and the PA spectra are shown in Figure 2 and Figure S1. The PA spectra were normalized at the photon energy of 3.7 eV. The PA spectra of TiO_2_ and TiO_2_/ZnS were almost the same and no apparent shift was observed. This is because the bandgap energy of ZnS (3.6 eV) was higher than that of TiO_2_ (3.2 eV). However, red shifts of the PA spectra were observed clearly after CdSe QD adsorption. Moreover, we found that the adsorption of the QD became faster in the case of the TiO_2_ electrode with ZnS passivation layers. This phenomenon is considered to result from the possible formation of CdS between ZnS and CdSe QDs. Therefore, the samples with and without ZnS passivation adsorbed CdSe QDs for 4 h and 9 h, respectively, to make the QD size the same. The results are displayed in Figure 2. Here, the PA shoulder was assumed to be the first excitation energy level, *E*_1_, of the CdSe QDs [9,21,22]. The average diameter of the QDs could be estimated by using the effective mass approximation [23,24], as shown in Equation (1):

(1) $$\Delta E=E_{1}-E_{g}=\frac{h^{2}}{8r^{2}}\left(\frac{1}{m_{e}}-\frac{1}{m_{h}}\right),$$

where Δ*E* is the bandgap shift, *E*_g_ is the bandgap energy, *r* is the radius of the QDs, and *m*_e_, *m*_h_, and *m*_0_ are the effective electron mass, hole mass, and electron rest mass, respectively (*m*_e_ = 0.13 m_0_, *m*_h_ = 0.44 *m*_0_, and *m*_0_ = 9.11 × 10^−31^ kg [25]). By applying the value of *E*_1_, the average diameter was estimated to be about 6.5 nm.

**Figure 3:**
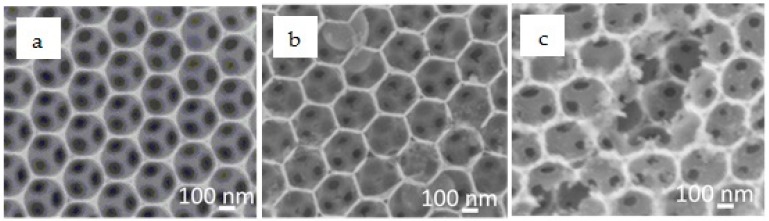
SEM images of inverse opal (IO)-TiO_2_ without ZnS passivation (**a**); with 5 cycles of ZnS passivation (**b**); with 15 cycles of ZnS passivation (**c**).

**Figure 4:**
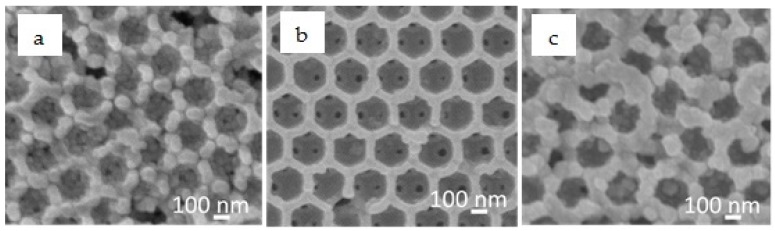
SEM images of CdSe QD deposited IO-TiO_2_ without ZnS passivation (**a**); with 5 cycles of ZnS passivation (**b**); with 15 cycles of ZnS passivation (**c**).

Figure 3 and Figure 4 show the typical scanning electron microscopy (SEM) images of the IO-TiO_2_ without ZnS passivation (a), with 5 cycles of ZnS passivation (b) and with 15 cycles of ZnS passivation (c), without and with CdSe QD adsorption, respectively. As shown in Figure 3, the wall of the IO-TiO_2_ electrode became thicker after ZnS passivation cycle increased. Figure 4 and Figure S2 show that CdSe QD formation initiated on the TiO_2_ surface. A highly ordered TiO_2_ framework with air holes was obtained after 5 cycles of ZnS passivation. However, at 15 cycles of ZnS passivation, the IO structure broke and its porous size became smaller after QD adsorption. The reason of the IO breakdown is the washing of the electrodes during the SILAR process. In this method, dissolved cationic and anionic precursors are put into two beakers, and then the bare electrode is dipped alternatively into each solution for growing the QDs on the electrode. After absorption of Zn^2+^ or S^2−^ ion, the electrode needs to be washed with methanol. With the number of ZnS deposition cycles increasing, washing time is multiply increasing. Therefore, it is thought that the surfaces of some IO structures were destroyed.

**Figure 5:**
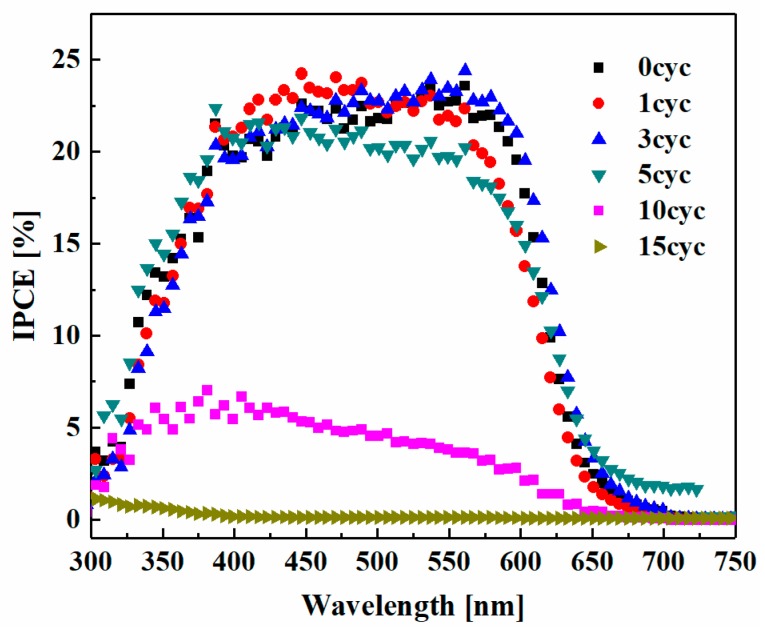
The incident photon-to-current conversion efficiency (IPCE) spectra for samples with different ZnS passivation layers.

Figure 5 shows the incident photon-to-current conversion efficiency (IPCE) spectra of the CdSe QDSSCs with different ZnS passivation layers. The IPCE peak value was about 24% at 550 nm for the device without ZnS passivation. For enhancing the IPCE values, it is necessary to prepare thicker IO-TiO_2_ electrodes. As shown in Figure 5, the IPCE value decreased greatly as the ZnS passivation cycle number exceeded 10. This is because of the reduction of electron injection from CdSe QDs to IO-TiO_2_ for thicker ZnS passivation layers.

To confirm the reduction of the electron injection for thicker ZnS passivation layers, transient absorption (TA) kinetics were detected for the CdSe QDs deposited on the IO-TiO_2_ with and without ZnS passivation. Figure S3 shows the TA responses of the samples without and with 10-cycle ZnS passivation. The probe wavelength used here was 570 nm, which corresponds to the band-edge absorption of the CdSe QDs. Therefore, the TA decay mostly reflects the electron injection from the CdSe QDs to the IO-TiO_2_. The TA response can be fitted using a bi-exponential function (*y* = *A*_1_exp(−*t*/*t*_1_) + *A*_2_exp(−*t*/*t*_2_) + *A*_3_)), and Table S1 shows the least-squares best fit parameters. From Figure S3 and Table S1, we can clearly find that the TA decay became much slower in the case of the samples with a 10-cycle ZnS passivation layer between the IO-TiO_2_ and the CdSe QDs. This result clearly indicated the reduction of electron injection from the CdSe QDs to IO-TiO_2_ for thicker ZnS passivation layers.

**Figure 6:**
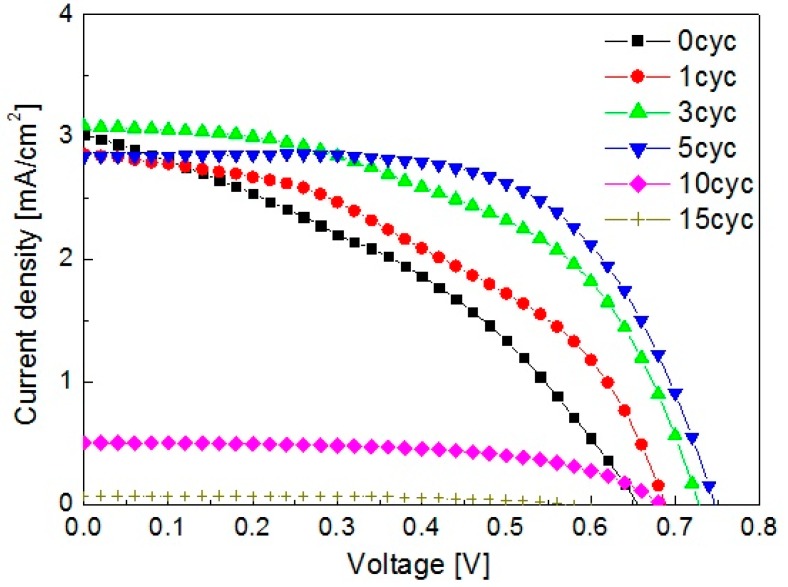
Photocurrent density–voltage *(J–V*) curves of CdSe quantum dot-sensitized solar cells (QDSSCs), where the IO-TiO_2_ is treated with (from 1 cycle to 15 cycles) and without ZnS surface passivation (0 cycle).

The dependence of the photocurrent density–voltage (*J*–*V*) properties on the passivation cycles is shown in Figure 6. The short-circuit current density (*J_sc_*), *V_oc_*, *FF*, and the power conversion efficiency (*η*) value of solar cells determined from *J–V* curves are summarized in Table 1. As the ZnS passivation cycle number increased up to 5, the *J_sc_* was almost unchanged, but *V_oc_* and *FF* increased greatly. Then, as the ZnS passivation cycle number increased further, i.e., the 10 and 15 cycles, *J_sc_* decreased significantly, which was consistent with the IPCE spectra. As a result, the solar cell with 5 cycles of ZnS passivation exhibited the highest *η* (1.33%). Compared to the cell without ZnS passivation, the presence of ZnS passivation layers significantly increased *η* by ~180%. *V_oc_* and *FF* were also remarkably enhanced with the increased ZnS passivation cycle up to 5 cycles. This result suggested that the ZnS passivation could reduce the interfacial recombination at the IO-TiO_2_/QDs and IO-TiO_2_/electrolyte interfaces, for which two possible explanations can be considered. One is the decrease of recombination at the IO-TiO_2_/electrolyte interface (Rec 2), and the other one is the reduction of the back-electron injection from the TiO_2_ electrode to QDs (Rec 3). On the other hand, the great reduction of *J_sc_* and IPCE values for 10 or more ZnS passivation cycles indicated that electron injection to TiO_2_ became very difficult for thicker ZnS passivation layers.

**Figure 7:**
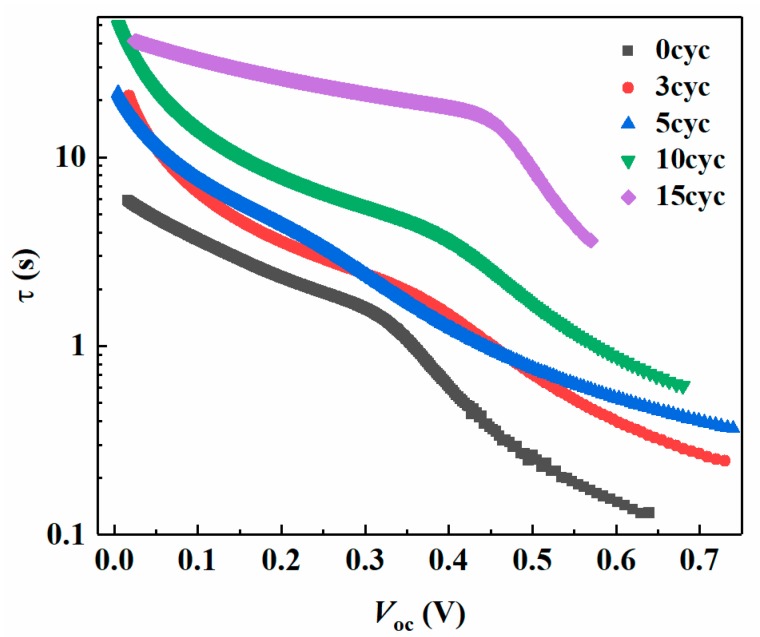
Effective electron lifetime curves of the IO-TiO_2_/CdSe QDSSCs, of which the IO-TiO_2_ electrodes were passivated with (from 1 cycle to 15 cycles) and without ZnS.

In order to reveal the effects of ZnS coating on charge carrier recombination and charge carrier lifetime in these QDSSCs, transient open-circuit voltage decay measurements (OCVD) were carried out. Figure S4 shows the OCVD curves of the IO-TiO_2_/CdSe QDSSCs, where the IO-TiO_2_ electrode was passivated with and without ZnS. The decay of *V_oc_* in the solar cells became slower with the increased ZnS passivation cycles. The effective electron lifetime (*τ*) from the decay of *V_oc_* can be determined by Equation (2) as follows [26,27]:

(2) $$\tau =\frac{k_{B}T}{e}{\left(\frac{dV_{OC}}{dt}\right)}^{-1}$$

where *e* is the elementary charge, *k_B_* is the Boltzmann constant and *T* is temperature. Figure 7 shows *τ* of CdSe QD-sensitized IO-TiO_2_ solar cells with different ZnS passivation cycles, which was calculated from the *V*_oc_ decay curves (Figure S4). From Figure 7, we can clearly see that *τ* of these QDSSCs with ZnS passivation layers was longer than that of device without ZnS passivation layers, especially at the high *V*_oc_ region. In addition, with the ZnS passivation cycle number increasing, the value of *τ* became larger at the same voltage. Larger *τ* reflected that there was less recombination in the devices. It means that both of recombination occurring at the IO-TiO_2_/electrolyte interface and the IO-TiO_2_/CdSe QDs interface were suppressed after ZnS coating. Notably, for 10-cycle and 15-cycle samples, although there was less recombination in these two type devices compared with the other four samples, the electron injection from CdSe QDs to IO-TiO_2_ became difficult due to the thicker ZnS layers, which resulted in low *J*_sc_ and efficiency. This result agreed strongly with the photovoltaic performance, as shown in Figure 6 and Table 1.

To clarify the mechanism of the ZnS passivation on the increase of the electron lifetime in the QDSSCs, impedance spectroscopy characterization was carried out. The results are shown in Figure S5. There was a large difference between the impedance spectra of the QDSSCs with and without ZnS passivation. Then, the recombination resistances, *R*_rec_, of the QDSSCs were obtained by fitting the impedance spectra (Figure S5a) with an equivalent circuit, according to the method reported by Bisquert and co-workers [28]. The ZnS passivation has a great effect on the *R*_rec_ and increased the *R*_rec_ greatly, as shown in Figure S5b. R_rec_ was reversely proportional to the recombination rate in the QDSSCs. The ZnS passivation on the IO-TiO_2_ electrodes decreased the recombination of the injected electrons in TiO_2_ to the holes remained in the CdSe QDs and/or to the accepting species in the electrolyte. Thus, as a result, *V*_oc_ and *FF* of the resultant QDSSCs were enhanced. This result is in good consistency with the OCVD results as mentioned above.

## 3. Materials and Methods

### 3.1. Preparation

The IO-TiO_2_ films were prepared on fluorine-doped tin oxide (FTO) coated glass by filling the void in an artificial template and subsequently removing the template [29,30]. Uniform polystyrene (PS) latex (the diameter was 474 nm) suspensions were dispersed ultrasonically for 30 min to break down the aggregated particles. The fabricated opal templates were prepared by vertically immersing the FTO substrate in a 0.1 wt.% PS suspension and fully evaporating the solvent in an oven at 40 °C for 1 to 2 days [29]. Subsequently, the substrate was immersed into a TiO_2_ precursor sol with a mixture of absolute ethanol, hydrochloric acid, tetrabutyl titanate, and deionized water to ensure the filling of all the voids for 10 min [30]. Finally, the substrate was annealed at 450 °C for 3 h in air with a ramp rate of 1 °C/min to remove the PS opal template. For the ZnS passivation on the IO-TiO_2_ surface, the SILAR method was used [19]. The IO-TiO_2_ electrode was immersed alternately in the Zn(CH_3_COO)_2_ solution (0.1 M in methanol) and the Na_2_S solution (0.1 M in pure water and methanol mixed solvent (1:1 by volume)) for 1 min. The processes were repeated several times (0, 1, 3, 5, 10, 15 cycles). After the ZnS passivation, the adsorption of CdSe QDs on IO-TiO_2_ was conducted at 10 °C by a chemical deposition method [31]. First, the Na_2_SeSO_3_ aqueous solution is prepared by stirring elemental Se powder in an aqueous solution of 200 mM Na_2_SO_3_ at 70 °C for about 6 h. Then, 80 mM CdSO_4_ and 120 mM N(CH_2_COONa)_3_ were mixed with an 80 mM Na_2_SeSO_3_ solution. The samples were immersed in the final chemical solution at 10 °C to promote the absorption of CdSe QDs and kept in the dark for some time (9 h for the sample without ZnS passivation and 4 h for those with ZnS passivation). After CdSe QD adsorption, the electrodes were passivated by ZnS using the SILAR method by 2 cycles to decrease the surface defects of the QD surfaces [9]. The thickness of the active layers (IO-TiO_2_/CdSe QD) without and with ZnS passivation on the IO-TiO_2_ was almost the same (about 6.5 μm), which were determined by the cross-sectional SEM images (Figure S6).

### 3.2. Measurement

The optical absorptions of the prepared electrodes were investigated using the PA technique [20]. The PA spectra were measured within the wavelength range between 300 and 800 nm at room temperature. The morphologies of samples were investigated with SEM (JSM-6340, JEOL, Japan) and TEM (JEM-2100F, JEOL, Japan). Sandwiched solar cells were assembled by using a CdSe QD-sensitized IO-TiO_2_ photoanode and a Cu_2_S counter electrode to measure the *J–V* characteristics, the IPCE spectra and the OCVD. A polysulfide solution was used as an electrolyte with the S and Na_2_S concentrations of 1 M [32,33]. The IPCE spectra were measured using a homemade equipment within the wavelength range between 300 and 800 nm. The *J–V* characteristics were measured using a solar simulator (Peccell Technologies, Inc., Yokohama, Japan) with 100 mW/cm^2^ under A.M. 1.5. The TA spectra were measured using a titanium/sapphire laser (CPA-2010, Clark-MXR, Inc., Dexter, MI, USA) with a repetition rate of 1 kHz and a pulse width of 150 fs. The wavelengths of the pump and probe light were 470 nm and 570 nm, respectively. The OCVD measurements were carried out using a 405 nm laser diode. The voltage decay from the device was recorded using a digital oscilloscope (DS-5554, Iwatsu, Japan). Electrochemical impedance spectra were measured under dark conditions using an impedance analyzer (SP-300, BioLogic, Seyssinet-Pariset, France), where a small voltage perturbation (10 mV rms) was applied at frequencies from 1 MHz to 0.1 Hz for different forward-biased voltages.

## 4. Conclusions

We have studied the effects of the passivation at the interface between IO-TiO_2_ and QDs with ZnS coating on the photovoltaic properties and its mechanism. *V_OC_* and *FF* have been improved to be as high as 0.74 V and 0.63, respectively, with 5 cycles of ZnS passivation. The efficiency of QDSSCs with IO-TiO_2_ electrodes was greatly improved (about 180%) after 5 cycles of ZnS passivation. The electron lifetime was enhanced significantly with the increased ZnS passivation cycle. However, excess ZnS passivation reduced electron injection from the QDs to the TiO_2_ for thicker ZnS passivation layers and worse penetration of electrolyte occurred due to the broken IO-TiO_2_ structure. In order to improve the photovoltaic properties of QDSSC with IO-TiO_2_ electrodes, it is needed to thicken TiO_2_ and optimize the passivation material and thickness. This work would shed light on the investigation of other multi-junction solar cells, such as perovskite solar cells.

## Figures and Tables

**Table 1:** Dependence of the photovoltaic properties on the passivation cycles ^a^.

ZnS Cycle	*J*_sc_ [mA/cm^2^]	*V*_oc_ [V]	*FF*	*η* [%]
0	3.0 ± 0.2	0.65 ± 0.01	0.37 ± 0.02	0.74 ± 0.02
1	2.9 ± 0.2	0.69 ± 0.02	0.44 ± 0.02	0.86 ± 0.05
3	3.1 ± 0.2	0.73 ± 0.01	0.52 ± 0.01	1.17 ± 0.03
5	2.8 ± 0.2	0.74 ± 0.01	0.63 ± 0.01	1.33 ± 0.03
10	0.5 ± 0.1	0.68 ± 0.02	0.58 ± 0.02	0.20 ± 0.04
15	0.1 ± 0.05	0.57 ± 0.03	0.57 ± 0.04	0.02 ± 0.01

^a^ To account for experimental errors, four devices of each type are measured to give the reported averages and deviations. The active area of each device is 0.28 cm^2^.

## Supplementary Materials

The following are available online at http://www.mdpi.com/2079-4991/8/7/460/s1, Figure S1: PA spectra of CdSe sensitized IO-TiO_2_ with and without ZnS passivation, Figure S2: TEM images of IO-TiO_2_, IO-TiO_2_/ZnS and IO-TiO_2_/ZnS/CdSe QD/ZnS, Figure S3: TA responses for the CdSe QD deposited IO-TiO_2_ with and without ZnS passivation, Figure S4: OCVD curves of IO-TiO_2_/CdSe QDSSCs, Figure S5: Impedance spectra of CdSe QDSSCs with and without ZnS passivation, Figure S6: Cross sectional SEM images of CdSe QD deposited IO-TiO_2_ with and without ZnS passivation, Table S1: Fitting result of Figure S3.
