# Supplementary material for: Interface Passivation Effects on the Photovoltaic Performance of Quantum Dot Sensitized Inverse Opal TiO2 Solar Cells
Source: Nanomaterials (Basel). 2018 Jun 25;8(7):460. doi: 10.3390/nano8070460 (PMC6071099; doi:10.3390/nano8070460)
Supplement: Supplementary file 1 [file nanomaterials-08-00460-s001.pdf]

Supporting Information

Article

Interface Passivation Effects on the Photovoltaic Performance of Quantum Dot Sensitized Inverse Opal TiO_2 Solar Cells

Kanae Hori[1], Yaohong Zhang[1,*], **Pimsiri Tusamalee**[1,2], **Naoki Nakazawa**[1], **Yasuha Yoshihara**[1], **Ruixiang Wang**[3], **Taro Toyoda**[1], **Shuzi Hayase**[4], **Qing Shen**[1,*]

[1] Department of Engineering Science, The University of Electro Communications, 1-5-1 Chofugaoka, Chofu, Tokyo 182-8585, Japan; hori@jupiter.pc.uec.ac.jp (K.H.); pimsiri.tsml@gmail.com (P.T.); nakazawa@jupiter.pc.uec.ac.jp (N.N.); yoshihara@jupiter.pc.uec.ac.jp (Y.Y.); toyoda@pc.uec.ac.jp (T.T.)

[2] Department of Physics, King Mongkut's Institute of Technology Ladkrabang, 1 Soi Chalongkrung 1, Ladkrabang, Bangkok 10520, Thailand

[3] Beijing Engineering Research Centre of Sustainable Energy and Buildings, Beijing University of Civil Engineering and Architecture, Beijing 102616, China; wangruixiang@bucea.edu.cn

[4] Graduate school of Life Science and Systems Engineering, Kyushu Institute of Technology, 2-4 Hibikino, Wakamatsu-ku, Kitakyushu, Fukuoka 808-0196, Japan; hayase@life.kyutech.ac.jp

***** Correspondence: yhzhang@jupiter.pc.uec.ac.jp (Y. Z.); shen@pc.uec.ac.jp (Q. S.); Tel.: +81-042-443-5471(Q. S.)

1. PA Spectra

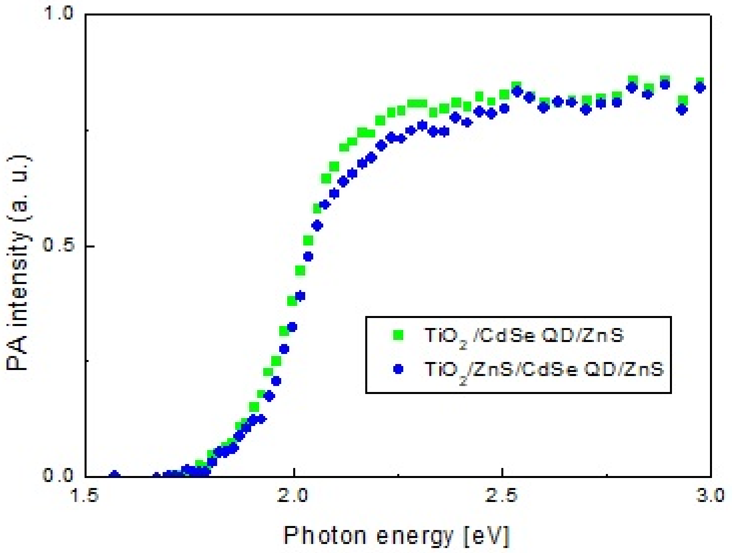

Figure S1. PA spectra of CdSe sensitized IO-TiO_2 with and without ZnS passivation.

2. Transmission electron microscopy (TEM) images

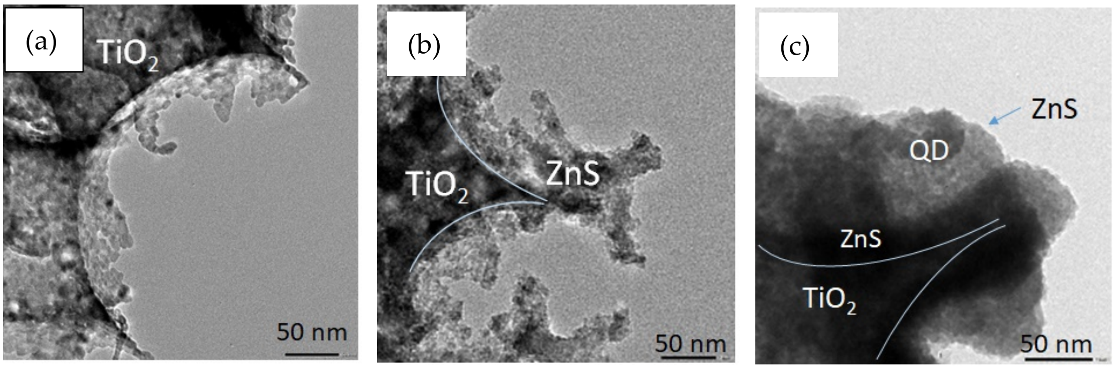

Figure S2. TEM images of IO-TiO_2 (a), IO-TiO_2/ZnS (b) and IO-TiO_2/ZnS/CdSe QD/ZnS (c).

3. Transient Absorption (TA) responses

Figure S3 shows the normalized TA responses with a probe light wavelength of 570 nm for the CdSe QD deposited IO-TiO_2 with and without ZnS passivation. Table S1 shows the least-squares best fit parameters of the TA signal of the CdSe QD deposited IO-TiO_2 with and without ZnS passivation.

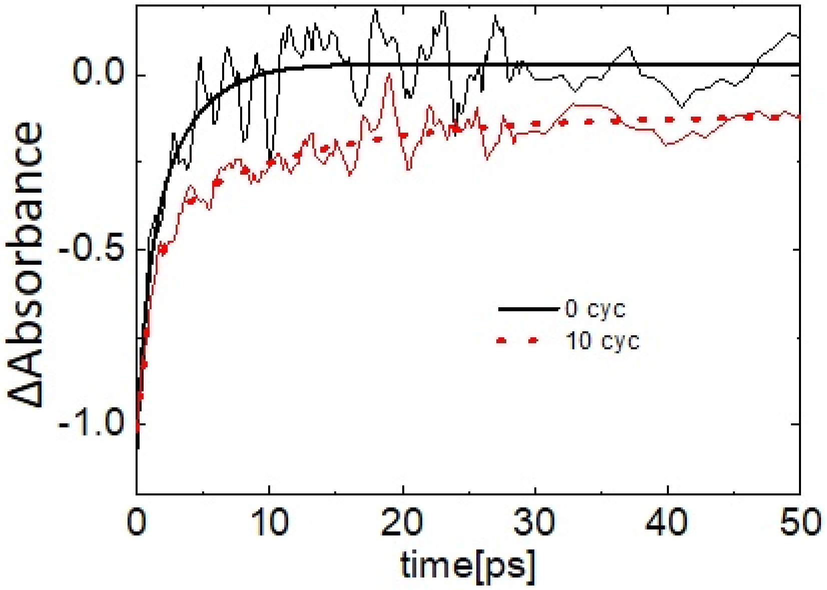

Figure S3. The normalized TA responses with a probe light wavelength of 570 nm for the CdSe QD deposited IO-TiO_2 with and without ZnS passivation.

Table S1. Fitted parameters of the TA kinetics with a bi-exponential function ($y=A_1exp(-t/t_1)+A_2exp(-t/t_2)+A_3)$) for CdSe QDs deposited on the IO-TiO_2 with and without ZnS passivation before the CdSe QD deposition.

ZnS cycle	A_1 [-]	A_2 [-]	A_3 [-]	t_1 [ps]	t_2 [ps]
0	-0.40	-0.63	0	0.73	3.06
10	-0.57	-0.31	-0.12	1.26	11.8

4. Transient open-circuit photovoltage decay measurement.

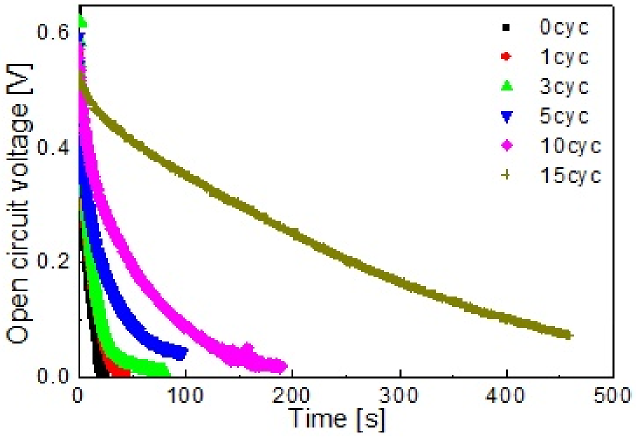

Figure S4. Open circuit voltage decay curves of the IO-TiO_2/CdSe QDSSCs, of which the IO-TiO_2 electrode was passivated with (from 1cycle to 15 cycle) and without ZnS.

5. Impedance spectroscopy characterization.

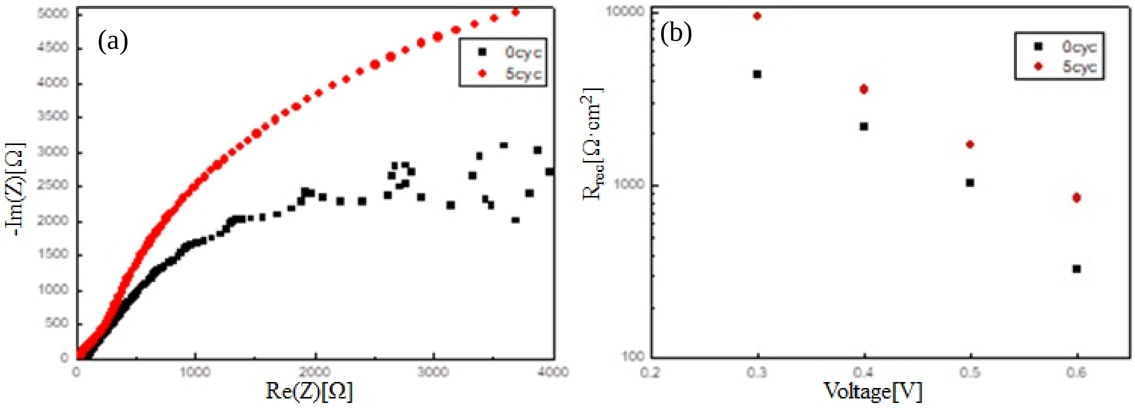

Figure S5. (a)Nyquist plot of CdSe QDSSCs with and without passivation.

(b) Recombination resistance R_{rec} obtained from the impedance spectra.

6. Cross sectional scanning electron microscopy (SEM) image

Figure S6 shows cross sectional SEM images of CdSe QD deposited IO-TiO2 with and without ZnS passivation.

(a) (b)

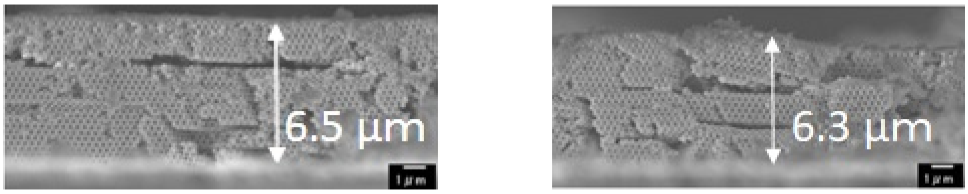

Figure S6. Cross sectional SEM images of CdSe QD deposited IO-TiO2 without (a) and with ZnS passivation (b).
